# Recycling of Flexible Polyurethane Foams by Regrinding Scraps into Powder to Replace Polyol for Re-Foaming

**DOI:** 10.3390/ma15176047

**Published:** 2022-09-01

**Authors:** Lei Guo, Wenchao Wang, Xiurui Guo, Kuanfa Hao, Haichao Liu, Yuan Xu, Gongxu Liu, Shouyun Guo, Lichen Bai, Donghui Ren, Fumin Liu

**Affiliations:** 1 College of Electromechanical Engineering, Qingdao University of Science & Technology, Qingdao 266061, China; 2 National Engineering Laboratory of Advanced Tire Equipment and Key Materials, Qingdao University of Science & Technology, Qingdao 266061, China; 3 Sino-Thai International Rubber College, Qingdao University of Science & Technology, Qingdao 266061, China

**Keywords:** flexible polyurethane foams, recycling, regrinding, powder, polyol replacement

## Abstract

In the context of protecting the ecological environment and carbon neutrality, high-value recycling of flexible polyurethane foam (F-PUF) scraps, generated in the production process, is of great significance to save petroleum raw materials and reduce energy consumption. In the present study, F-PUF scraps were ground into powder by strong shear regrinding using two-roll mill and then reused as a partial replacement of polyol for re-foaming. A series of characterizations were employed to investigate the effect of milling cycles, roller temperatures, and content of the powder on the properties of the powder and F-PUF containing powder. It was revealed that the mechanochemical effect induced breaking of the cross-linking structure and increased activity of the powder. The volume mean diameter (VMD) of powder prepared with 7 milling cycles, at room temperature, is about 97.73 μm. The microstructure and density of the F-PUF containing powder prepared in the above-mentioned manner to replace up to 15 wt.% polyol, is similar to the original F-PUF, with resilience 49.08% and compression set 7.8%, which indicates that the recycling method will play an important role in industrial applications.

## 1. Introduction

**Figure 1:**
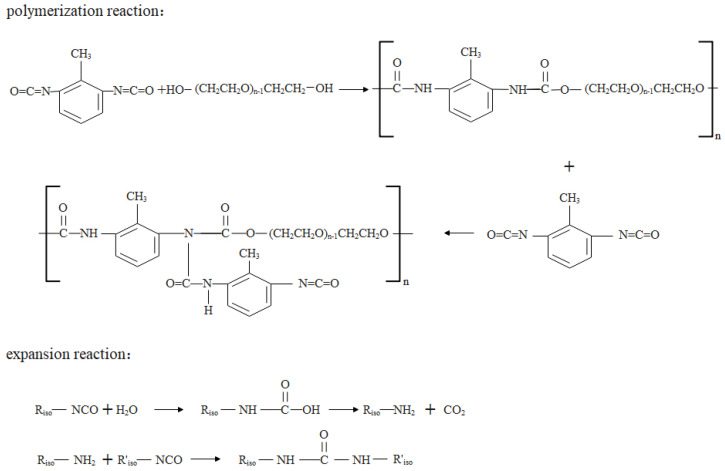
Scheme of F-PUF polymerization and expansion.

As one of the most commercially important specialty polymers, polyurethanes are widely used in everyday life, and among the 20 million tons of polyurethane produced annually in the world, polyurethane foam (PUF) accounts for 68% [1,2]. PUF is mainly classified into two types: flexible polyurethane foam (F-PUF), and rigid foam (R-PUF). F-PUF has a nearly complete open cell structure with densities as low as 20 kg/m^3^; while R-PUF is mostly of closed cell structure with bulk densities typically between 30 and 35 kg/m^3^. Among them, F-PUF accounts for more than half of the foam market [3] and has been applied widely in filters, automotive, and furniture/bedding cushioning [4]. However, a large number of F-PUF scraps are produced, accompanied by high output. It is estimated that the scraps account for 10% of product volume during the production process of F-PUF [5]. As shown in Figure 1, the preparation process of F-PUF mainly includes two reactions: polymerization reaction, and expansion reaction. Among them, the polymerization reaction between polyisocyanates and polyether polyol makes F-PUF a kind of highly crosslinked thermosetting polymer material, containing carbamate groups, urea groups, and so on, which results in difficulties with recycling [6]. At present, landfill and incineration are still widely used as treatment methods for F-PUF scraps and wastes [7,8]. However, F-PUF is difficult to degrade and harmful gases such as isocyanate fumes, hydrocyanic acid, or nitrogen oxides, will be generated during the incineration process [9,10]. Therefore, neither landfill nor incineration is acceptable, considering the long-range ecological goals of zero pollution. In the context of carbon neutrality, high-value recycling of these considerable scraps has become more and more attractive.

There are two major recycling methods for F-PUF scraps: chemical recycling, and mechanical recycling. Chemical recycling makes the reaction of the functional groups, such as carbamate in the F-PUFs to depolymerize the main chain, and aims to recycle the initial feedstock, especially polyol, which can be reused in foam production [11]. Existing chemical recycling methods mainly include acidolysis [12,13], glycolysis [14,15], hydrolysis [16], and aminolysis [17]. Among them, since the hydrolysis is carried out under high temperature and high pressure, the requirements for reaction conditions and equipment are very high, and the separation and purification of hydrolytic products of PU are, so far, costly, making the process economically unattractive [12]. The main product of aminolysis contains aromatic amines, which are classified as carcinogenic, such as toluene diamine and methylene diamine. In contrast to the two methods mentioned above, the main product of glycolysis has a lower content of primary aromatic amine; acidolysis does not lead to the formation of any primary aromatic amine. More importantly, both acidolysis products and glycolysis products can be used to prepare polyurethane. Therefore, the most commonly used methods are acidolysis and glycolysis, which can be carried out on an industrial scale [1]. However, the product of chemical recycling is a mixture of polyol, amines, and other impurities, which are difficult to fractionate and purify [18]. Moreover, the equipment and processes used in industrial production are complex, which leads to high investment costs and low added value for general F-PUF manufacturers [2,19]. Mechanical recycling aims to reuse F-PUF scraps with physical treatment. The scraps are usually crushed into small particles to be used as fillers, or bonded and compressed into other products. Compared with chemical recycling, mechanical recycling is more economical and easier to achieve [1]. Existing mechanical recycling methods mainly include rebonding [20,21], adhesive pressing [22], compression molding [23], injection molding [24], and regrinding [25]. Among these methods, regrinding is the most attractive mechanical method due to its high economic value. The recycling method involves two steps: grinding F-PUF scraps into fine powder, and mixing the powder with the polyol for re-foaming. To ensure the powder can be used as fillers in PUF, the particle size should be less than the average wall thickness of PUF products, about 200 μm [26], so that the powder can be incorporated in the cell walls without affecting the overall cell structure. Different technologies are used to reduce the particle size of F-PUF scraps; the main techniques are cutting and milling [27]. Unlike R-PUF, F-PUF is relatively soft and has higher elasticity at room temperature. The glass transition temperature of F-PUF is about −50 °C [28], which causes cutting methods such as precision-knife-cutting difficult to meet the requirements of powder preparation at room temperature. In contrast, milling methods such as pellet milling, and two-roll milling, are more suitable for the grinding of F-PUF. According to the literature [27], the particle size of the powder obtained by two-roll milling is the smallest, which can reach below 100 μm; there are already application cases of regrinding technology. It is stated that F-PUF powder can be incorporated in amounts between 15 and 25 wt.% into molded foam car seats [26]. Bayer Corporation has also claimed that they had invented a method for making energy-absorbing foam with PU fillers [20]. However, the decrease of mechanical properties of re-foaming products limits the application of regrinding [27]. In the research mentioned above, regrinding is considered to involve only physical action, and F-PUF scraps are reused as passive filler. However, it was found that R-PUF scraps showed certain activity after being crushed into powder by regrinding [28], and the powder made by two-roll milling was used as a polyol replacement in polyurethane adhesives; but the mechanism of replacing polyol with powder was not mentioned. We also found that the F-PUF powder made by two-roll milling showed certain activity. However, to the best of the authors’ knowledge, little research has been reported on the activation mechanism of the powder.

In the present study, F-PUF scraps were ground into powder by strong shear regrinding using two-roll mill, and the activation mechanism of the prepared powder was analyzed. To verify the activation mechanism, the prepared powder was then used as the replacement for polyol in the re-foaming process. To characterize the properties and the potential activity of the powder under different regrinding conditions, the particle size, particle temperature, morphologies, and Fourier transform infrared spectroscopy (FTIR), as well as X-ray photoelectron spectroscopy (XPS) of the powder, were investigated. The morphologies, FTIR, macro quality (including density, resilience, and compression set) of F-PUF containing powder, and the original F-PUF, were compared to evaluate the effect of the powder on the re-foaming products.

## 2. Materials and Methods

### 2.1. Materials

F-PUF scraps used to recycle: super soft sponge (density 24 kg/m^3^, resilience 50%, compression set 7.2%, Man Wah Holdings Limited, Hong Kong, China).

Chemicals used in F-PUF preparation (re-foaming): toluene diisocyanate (TDI, Wanhua Chemical Group Co., Ltd., Yantai, China), polyether polyol (5616s, viscosity 980 mPa.s, OHN 56 mg KOH/g, Wanhua Chemical Group Co., Ltd., Yantai, China), deionized water, dichloromethane (MC, Changhua Chemical Technology Co., Ltd., Suzhou, China), triethylenediamine (A33, mixed with 80% 5616s, Changhua Chemical Technology Co., Ltd., Suzhou, China), divalent tin (T9, mixed with 80% 5616s, Wanhua Chemical Group Co., Ltd., Yantai, China), silicone oil (L618, mixed with 80% 5616s, Huizhou Yuanan New Material Co., Ltd., Huizhou, China), and amine accelerator (A1, Changhua Chemical Technology Co., Ltd., Suzhou, China).

### 2.2. Preparation of Samples

**Figure 2:**
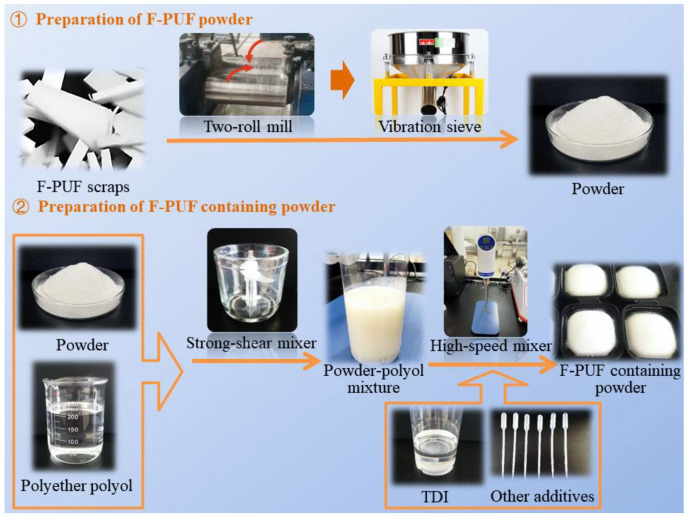
Schematic diagram of the recycling process.

There are two steps in the recycling process of F-PUF scraps: the preparation of the powder and the preparation of F-PUF containing powder, as shown in Figure 2.

#### 2.2.1. Preparation of F-PUF Powder

F-PUF scraps were initially crushed into strips approximately 20 cm long, 10 cm wide, and 2 cm thick, for subsequent regrinding. The two-roll mill X(S)K-160 of Shanghai Rubber Machinery First Factory Co., Ltd. (Shanghai, China) was used as the powder preparation equipment. In order to prepare micron-level powder, the roller pitch was adjusted to minimum and the speed ratio of the two rollers was set to 1:1.2. The control variate method was adopted to investigate the influence of milling cycles and roller temperatures on the performance of the powder. A series of powder samples were prepared with four kinds of milling cycles (3, 5, 7, and 9), at room temperature, to study the influence of the number of milling cycles. Another series of powder samples were prepared with 7 milling cycles under four kinds of roller temperatures (room temperature, 40 °C, 60 °C, and 80 °C) for a roller temperature influence study. Finally, all the powder samples were sieved to ensure that the particle size was less than 200 μm.

#### 2.2.2. Preparation of F-PUF Containing Powder

In order to analyze the performance of F-PUF containing powder, a re-foaming process was carried out. The powder was firstly mixed with the polyether polyol by the strong-shear mixer ZG-J208C of Ningbo Zhaoji Electric Co., Ltd. (Ningbo, China). The speed of the mixer was set to 2000 r/min, and the mixing time was 30 min. Four different powder mass contents in the powder-polyol mixture (5%, 10%, 15%, and 20%) were adopted to study the effect of powder content on the F-PUF products. The preparation formula of the F-PUF containing powder is shown in Table 1. The one-step method was obtained for re-foaming as described: the raw materials are weighed according to the formula; then, the powder-polyol mixture and other additives were mixed with the high-speed mixer SN-OES-200SH of Shanghai Shangpu Instrument Equipment Co., Ltd. (Shanghai, China), and TDI was added to the mixture. After the components were evenly mixed, the mixture was quickly injected into the carton mold for foaming; finally, the foam sample was placed for 24 h to mature.

### 2.3. Measurements and Characterization

#### 2.3.1. Characterization of Powder

Particle size measurements were performed on the instrument Laser granulometer HELOS (H3839) & SUCELL2, R5 (SYMPATEC GmbH, Clausthal-Zellerfeld, Germany) with a measuring range 0.1–875 μm in the wet state. The powder was dispersed in deionized water by stirring before the measurements. The VMD was automatically calculated with output through PAQXOS 3.0.2 software. Each sample was measured 10 times and the mean of the 10 VMD values was calculated as the recorded value.

The particle temperature of powder during the regrinding process was measured by infrared thermometer FLUKE 62 MAX (Fluke Testing Instruments (Shanghai) Co., Ltd., Shanghai, China). Each sample was measured three times and the mean of the three values was calculated as the recorded value.

The morphologies of powder were examined by scanning electron microscopy (SEM) using JSM-7500F (JEOL Ltd., Tokyo, Japan). First, the powder was dispersed in deionized water. Then, it was coated on a cover glass to dry, and vacuum gold coated before SEM imaging.

The FTIR of powder was recorded with an EQUINOX 55 spectrometer (FTIR, Bruker Co., Bremen, Germany) in the range of 4000–500 cm^−1^ with a universal attenuated total reflectance sensor (ATR). The spectra were used to determine functional groups on the molecular structures of the powder.

The surface’s chemical composition of powder was assessed by XPS analyses. The XPS spectra were obtained by AXIS Supra (Shimadzu Co., Kyoto, Japan) using monochromatic Al/Kα radiation (100 W, *hυ* = 1486.6 eV), and the unique set of binding energy of each element was used to identify and determine the concentration of elements on the surface.

#### 2.3.2. Characterization of F-PUF Containing Powder

The morphologies of F-PUF containing powder were examined by the same SEM as used for the powder observation. The samples were first cryogenically fractured in liquid nitrogen to produce a freshly fractured cross-sectional surface. Before SEM imaging, the samples were sputtered with a thin layer of gold under vacuum. 

The FTIR and XPS of F-PUF containing powder were measured in the same way as that of the powder.

The density of F-PUF containing powder was determined by calculation of the mass and volume. The samples used in the density test were determined according to the standard ASTMD D3574-17, and the mass of the samples was measured by high precision electronic balance JA3003C (Shanghai Yueping scientific Instrument Co., Ltd., Shanghai, China). Each sample was measured three times and the mean of the three values was calculated as the recorded value.

The resilience of F-PUF containing powder was determined according to Chinese national standard GB/T 6670-1997 with a ball rebound tester PMLQ-500 (Beijing Guance Jingdian Equipment Co., Ltd., Beijing, China). Each sample was measured three times and the mean of the three values was calculated as the recorded value.

The compression set of F-PUF containing powder was determined according to Chinese national standard GB/T 6669-2001 with a compression set tester YSBX-1 (Beijing Guance Jingdian Equipment Co., Ltd., Beijing, China). Each sample was measured three times and the mean of the three values was calculated as the recorded value.

## 3. Results and Discussion

### 3.1. The Activation Mechanism of the Powder

During the regrinding process, the two rollers rotate in opposite directions and at different speeds to generate strong, shear extrusion and tensile forces in the very narrow gap between them, which act on polyurethane segments to produce extremely high internal stress. So, it can be assumed that the cross-linking structure in the F-PUF is destroyed under the action of internal stress. Actually, the cross-linking structure in F-PUF is classified into two types: physical cross-linking, and chemical cross-linking. 

Physical cross-linking is the result of hydrogen bonds. Hydrogen bonds exist between the groups containing the electronegative nitrogen atom, or oxygen atom, and the groups containing the hydrogen atom. Most of the imine (-NH) groups in polyurethane can form hydrogen bonds, and most of them are formed by -NH groups and the carbonyl groups in the hard segment; a small part is related to the ether oxygen or ester in the soft segment [29]. In the regrinding process, the hydrogen bonds are broken due to the mechanochemical effect; thereby, the cross-linking between the polyurethane molecular chains is weakened and the surface activity of the powder is improved. 

**Figure 3:**
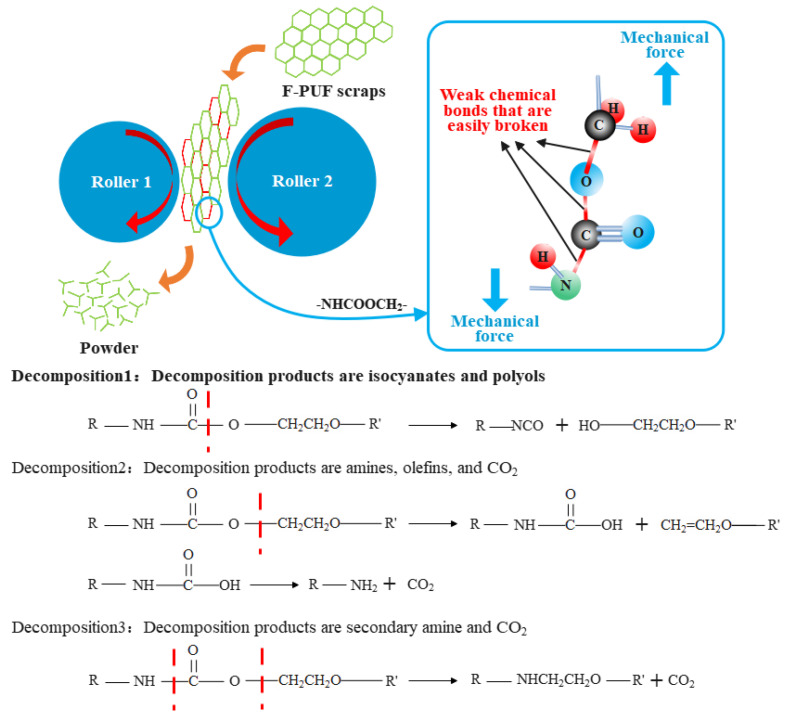
Scheme of chemical crosslinking decomposition reaction of F-PUF in the regrinding process.

Chemical cross-linking is formed by chemical bonds. The mechanochemical effect during the regrinding process increases the molecular chain’s internal energy and breaks the chemical bonds with weaker bond energy. The initial temperature of the thermal decomposition of each kind of group in the polyurethane main chain can be used to infer the position where the chemical bonds are easily broken during the rebinding process. It is reported that the initial temperature of thermal decomposition of the groups in the polyurethane main chain is: 100–120 °C for allophanate groups, 115–125 °C for biuret groups, 140–160 °C for carbamate groups, and 160–200 °C for urea groups [30]. Generally, the thermal degradation of allophanate and biuret is reversible, and decomposition products are mainly carbamate and urea. The thermal degradation temperature of carbamate is lower than urea, so the groups that are the most easily broken on the main chain are the carbamate groups; the breaking position of the chemical bonds in the polyurethane groups is also related to the bond energy and polarity. The common chemical bond energy of the chemical bonds in carbamate groups is 332 kJ/mol for C-C bonds, 326 kJ/mol for C-O bonds, and 305 kJ/mol for C-N bonds. The C-O bonds are more polar than the C-N bonds; therefore, we can speculate that the C-O bonds and C-N bonds in the carbamate groups are not difficult to break. Based on the above analysis, we can assume that the chemical cross-linking cleavage reaction of the F-PUF may fall into the three categories shown in Figure 3. Among them, the isocyanate (-NCO) groups in the products of decomposition 1 have high reactivity and may immediately react with the moisture present in the air to form CO_2_ gas and amine groups [31], and react with carboxylic acid to generate amides under hot oxygen conditions simultaneously [32]. However, the -OH groups generated will be retained; therefore, the -OH groups in the products of decomposition 1 can be used to replace part of the polyol in the re-foaming raw materials.

In addition, even though the isocyanate index is greater than 1.0 in most F-PUF production formulation, some -OH groups may remain in F-PUF products [33]. This is because the reactivity of the primary and secondary hydroxyl groups is differentiated due to the effects of steric hindrance [34], and part of the -OH groups are not involved in the reaction, which is verified in the follow-up XPS analysis. The regrinding process may weaken the steric hindrance and increase the reactivity of some -OH groups.

### 3.2. Properties of the Powder

#### 3.2.1. Particle Size and Particle Temperature

**Figure 4:**
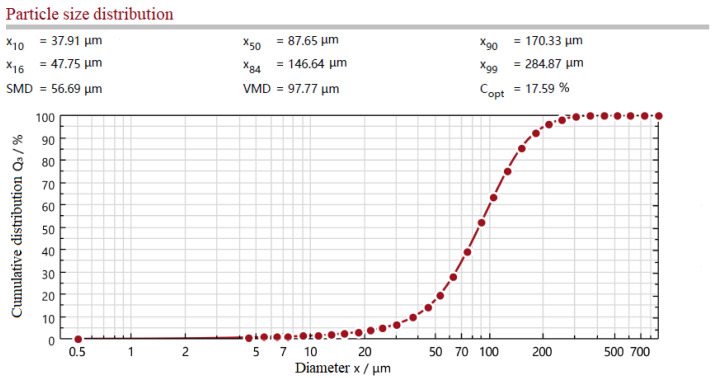
Single test result of particle size distribution of the powder prepared in Experiment 3.

As described in Section 3.1, the activity of the particles mainly originates from the destruction of the cross-linking structure caused by internal stress. Both temperature and mechanical force can lead to the destruction of the cross-linking structure and reduction of the particle size of the powder. The milling cycles represent the strength of the applied mechanical force; therefore, temperature and milling cycles are two important factors in reducing particle size. In order to analyze the effect of milling cycles and roller temperatures on the particle size, the particle size distribution and the particle temperature of the powder obtained under different regrinding conditions, are demonstrated in Table 2. It should be noted that although the milling process generates heat, since the interval time between two milling cycles is much longer than the milling time and the max milling cycles is only 9, milling cycles have little effect on the roller temperature with the action of the temperature control system; the roller temperature depends on the temperature control system settings. It can be seen that the VMD of the powder decreases with an increase of milling cycles when the roller temperature is room temperature (Experiment 1–4). When the milling cycles reach 7 or more, the particle size of 90% powder can be guaranteed to be less than 200 μm, as shown in Figure 4, which indicates that most of the prepared powder meets the particle size requirement [26] and can be used in the subsequent re-foaming process. Since the interval time is much longer than the milling time, the powder can be cooled between two-roller milling cycles. As a result, milling cycles have little effect on the particle temperature, and the temperature of the powder obtained in the experiment of different milling cycles is between about 70–80 °C. As for the effect of roller temperatures, it is manifest from Table 2 (Experiment 3, 5–7) that increasing the roller temperature can also reduce the particle size, as well as lead to the increase of particle temperature. However, in the experimental roller temperature range, the effect of roller temperatures on particle size is less than milling cycles. It should be mentioned that all particle temperatures of the powder are less than 110 °C, which is considered to be the initial dissociation temperature of common F-PUF [2,35]. Therefore, the reduction of particle size is mainly due to the strong shearing mechanical force during the regrinding process.

#### 3.2.2. Morphologies

**Figure 5:**
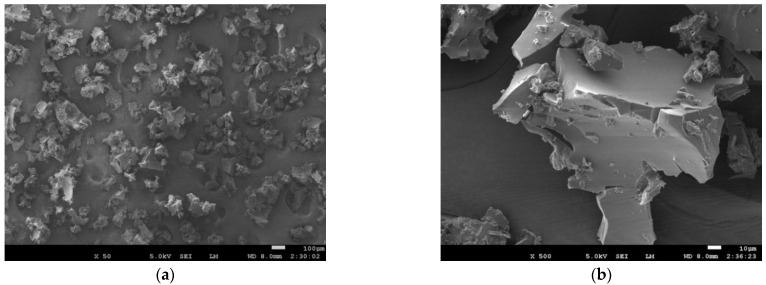
SEM photographs of the powder: (**a**) mag. ×50, (**b**) mag. ×500.

Microscopic morphologies of the prepared powder are provided to analyze the physical structure of the powder. Since the physical structure of the powder obtained in all the experiments is similar, the powder obtained in Experiment 3 is taken as an example for analysis, and the microscopic morphologies of magnification at 50 times and 500 times are shown in Figure 5. It is further confirmed that the particle size is relatively average in Figure 5a. The particle shape shown in Figure 5b is complex and irregular, and the cell structure of F-PUF is no longer visible, which is beneficial to increase the contact area with polyol in the subsequent mixing process and to enhance the mixing effects.

#### 3.2.3. FTIR Analysis

**Figure 6:**
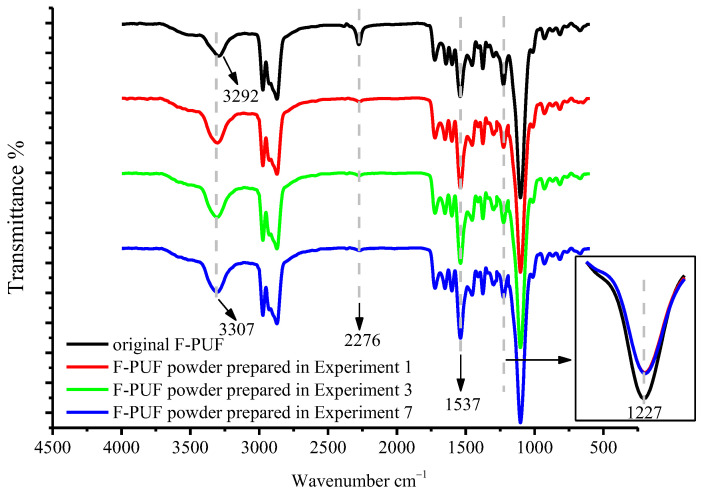
ATR-FTIR spectra of the powder and the original F-PUF.

To illustrate the powder activity from the chemical structure, the ATR-FTIR spectra of the powder prepared in Experiments 1, 3, and 7, and the original F-PUF, are utilized, as shown in Figure 6. The peaks at 1227, 1537, 2276, and 3292 (or 3307) cm^−1^ in all four samples are assigned to the stretching vibration of C-O bonds in ester groups, amide II, -NCO groups and -NH groups (or -OH groups), respectively. ATR-FTIR spectra of the prepared powder under different conditions are similar, while significantly different peak intensity can be seen at 1227, 2276, and 3292 (or 3307) cm^−1^ between the infrared absorption of the powder and the original F-PUF, which means that mechanochemical reactions do occur during the regrinding process and the chemical structure is changed. Compared with the peak intensity at 3292 cm^−1^ of the original F-PUF, the peak intensity of all three powder samples is increased and shift to 3307 cm^−1^. This may be due to the breaking of the hydrogen bonds formed by -NH groups, or the primary amines decomposed in the Decomposition 1 and the Decomposition 2, or the -OH groups decomposed in Decomposition 1. The peak intensity of the powder is decreased at 1227 cm^−1^ compared with that of the original F-PUF, which indicates the breakage of C-O in ester groups, and can be caused by all the three decompositions similar to the thermal degradation of polyurethane [2]. -NCO groups remaining in the original F-PUF, and decomposed in Decomposition 1, may react with moisture and carboxylic acid to generate amine groups and amides under hot oxygen conditions, which lead to the decrease in peak intensity at 2276 cm^−1^. The amide groups and amine groups in the reaction product enhance the vibration peaks at 1537 and 3307 cm^−1^. Since there are almost no -NCO groups in the powder, the -OH groups that may be generated in the regrinding process can be retained, and the hydrogen bonds formed by -NH groups may also be broken, causing an increase in the surface activity of the powder.

#### 3.2.4. XPS Analysis

**Figure 7:**
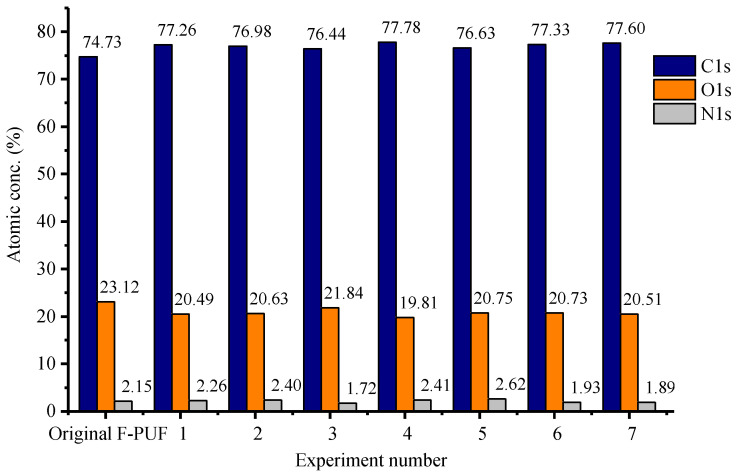
Surface compositions of the powder and original F-PUF.

To further confirm the generation of activated groups on the powder surface, the XPS measurement of the prepared powder and the original F-PUF is performed. According to the surface compositions reported in Figure 7, the content of C1s is increased and the content of O1s is decreased after the regrinding process. This may be due to the production of CO_2_ in the decompositions, which leads to a relative decrease in the content of the oxygen element. However, there is no significant regularity between the regrinding conditions and the surface compositions.

**Figure 8:**
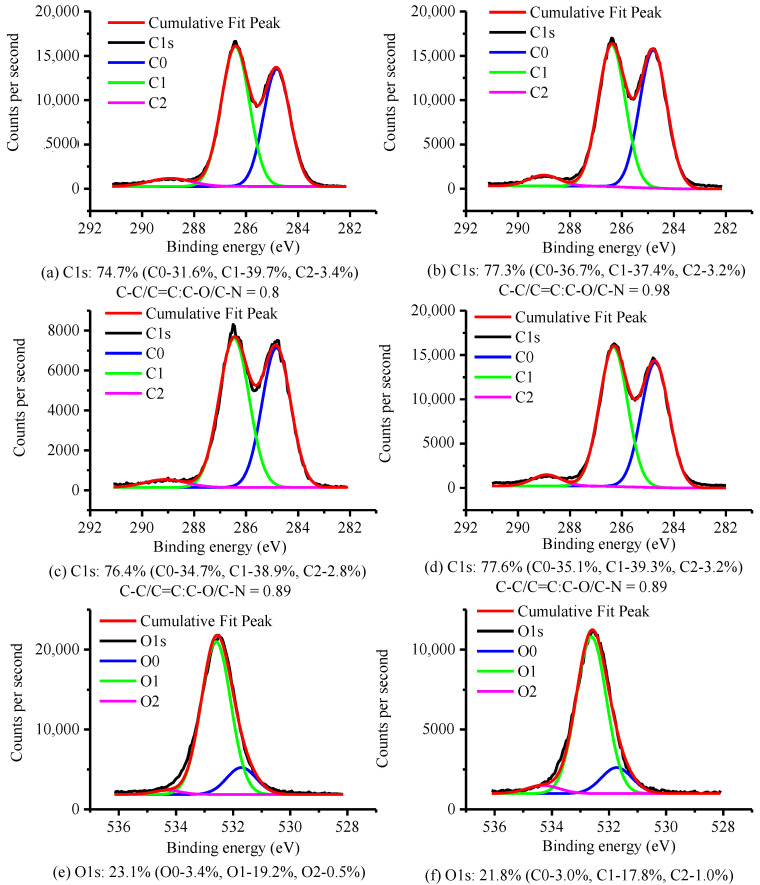
High-resolution C1s XPS images of: (**a**) the original F-PUF, (**b**–**d**) the powder prepared in Experiment 1, 3, and 7; high-resolution O1s XPS images of; (**e**) original F-PUF; (**f**) the powder prepared in Experiment 3.

High-resolution C1s XPS images of the powder prepared in Experiments 1, 3, and 7, and the original F-PUF, are shown in Figure 8a–d. The C0 fraction at 284.8 eV is associated with C-C/C=C bonds, the C1 at 286.4 eV represents C-O/C-N bonds, and C=O bonds are indicated by the C2 peak at 289 eV [36,37]. The ratio of C-C/C=C bonds content to C-O/C-N bonds content (C-C/C=C: C-O/C-N) is also provided in Figure 8. It is shown that C-C/C=C: C-O/C-N increases after regrinding, proving that there are C-O/C-N bonds broken. Considering that the C-O bonds are more polar than the C-N bonds, and combined with the results of ATR-FTIR analysis, it can be confirmed that the C-O bonds are broken during the regrinding process.

To further analyze the groups generated by C-O bonds breakage, Figure 8e–f shows the O1s high-resolution spectra of the powder prepared in Experiment 3, and the original F-PUF. The O0 fraction at 531.7 eV is associated with carbonyl groups, the O1 at 532.6 eV represents ether-type oxygen, and the O-H linkages are indicated by the O2 peak at 534.3 eV [38,39]. We can see that the content of O2 is approximately equal to the content of C2, both of which refer to the content of C=O, which indicates the correctness of the peak fitting process of the O1s spectrum. It is obvious that there are -OH groups in the original F-PUF, which is consistent with our speculation. By comparing the two figures, it can be seen that although the oxygen content decreases due to the production of CO_2_ after the regrinding process, the content of O2 increases, which indicates that new -OH groups have been generated on the powder surface.

### 3.3. Properties of F-PUF Containing Powder

#### 3.3.1. Morphologies

**Figure 9:**
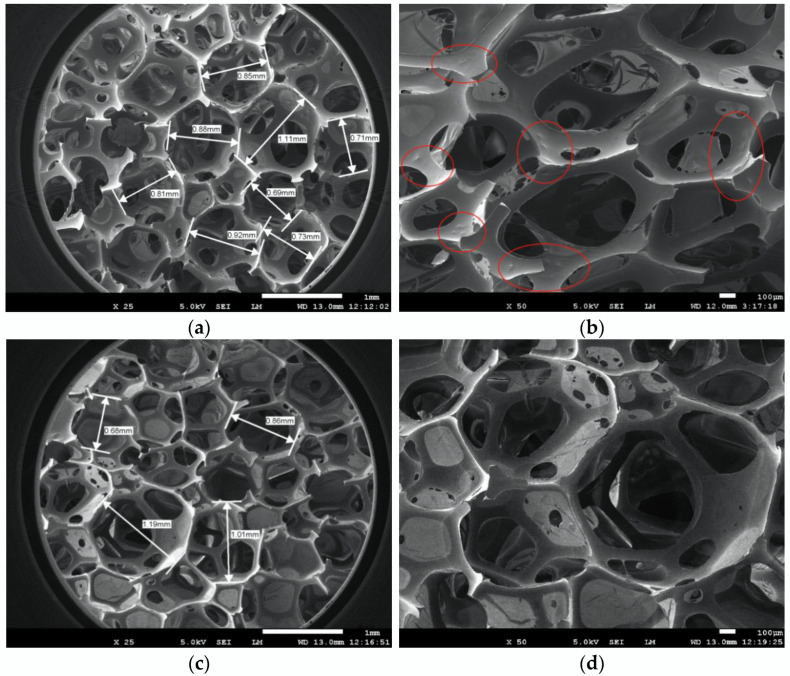
SEM photographs of the F-PUF containing powder (15% in the powder-polyol mixture) prepared in Experiment 3: (**a**) at ×25; (**b**) ×50; (**c**) original F-PUF at ×25; (**d**) ×50.

The microscopic morphologies of F-PUF containing powder (15% in the powder-polyol mixture) prepared in Experiment 3 and original F-PUF at two magnifications are shown in Figure 9. At magnification ×25, the complex porous structure of the F-PUF containing powder is similar to the observed structure of the original F-PUF. And there is no significant difference in cell size and cell wall thickness. However, at magnification ×50, many small depressions, which are rarely seen in the original F-PUF, are observed on the cell wall of the F-PUF containing powder, as shown by the red circle in Figure 9b. This is because the -OH groups involved in the extension reaction of the re-foaming process only exist on the surface of the powder, while the inside of the powder is still in a solidified state, resulting in small tensile deformation during the foaming process and the final formation of depressions.

#### 3.3.2. FTIR Analysis

**Figure 10:**
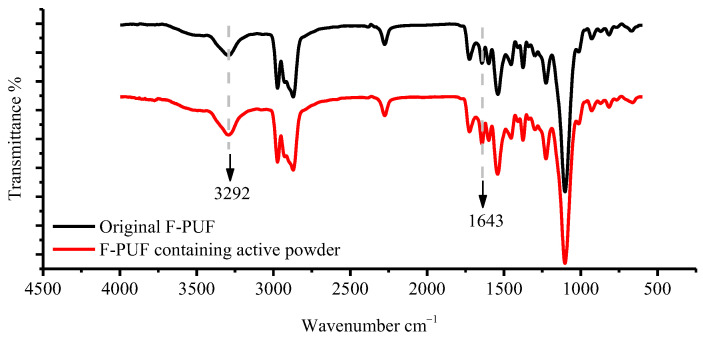
ATR-FTIR spectra of the F-PUF containing powder (15% in powder-polyol mixture) prepared in Experiment 3 and original F-PUF.

In order to clarify the modification of chemical structures of the F-PUF after adding powder, the ATR-FTIR spectra of the F-PUF containing powder (15% in the powder-polyol mixture) prepared in Experiment 3, and the original F-PUF, are compared in Figure 10. It can be seen that although a slight excess of isocyanate leads to a slight increase in the intensity of the peak representing urea groups at 1643 cm^−1^, and the peak representing -NH groups at 3292 cm^−1^, the FTIR spectra of the F-PUF containing powder are similar to those of the original F-PUF, which indicates that the powder can replace polyol to participate in the polymerization reaction during the re-foaming process.

#### 3.3.3. Density, Resilience and Compression Set

For evaluating the effect of regrinding conditions, powder content and microscopic depression on the macro quality of the F-PUF containing powder, the density, resilience, and compression set were measured, and compared with the original F-PUF, as shown in Table 3. It can be observed that the F-PUF containing powder prepared in Experiment 3 to replace up to 5 wt.% polyol has the best macroscopic quality, with resilience 50.08% and the compression set 7.2%, which is similar to the original F-PUF. As for the influence caused by regrinding conditions, it can be seen that increasing milling cycles can slightly reduce the density of F-PUF containing powder. This is due to the fact that the more milling cycles, the smaller the particle size of the powder, and the weaker the local inhibitory effect on foam expansion it plays in the re-foaming process, which leads to a relative increase in the PUF’s volume. However, in the experimental roller temperature range, the effect of roller temperatures is not obvious. As for the influence of the powder content, we can see that the addition of the powder has little effect on the density of the products, and only causes a slight decrease in resilience and increase in the compression set, but it is still within the range of product requirements (resilience > 35%, compression set < 8% according to Chinese national standard GB/T 10802-2006) when the powder content in the powder-polyol mixture is within 15%. Among the PUF containing powder to replace 15 wt.% polyol, the macro quality of the sample prepared in Experiment 3 has the best macroscopic quality, with resilience 49.08% and the compression set 7.8%. This means that the F-PUF containing powder prepared by us meets the product quality inspection requirements.

## 4. Conclusions

F-PUF scraps can be recycled into powder by regrinding using two-roll mill, and then reused as a partial replacement of polyol for re-foaming. This is due to the mechanochemical decomposition during the regrinding process to generate new -OH groups and the increase in the activity of the unreacted -OH groups in the original F-PUF. The hydrogen bonds formed by -NH groups may also be broken, causing an increase in the surface activity of the powder. 

Experiments show that the F-PUF containing powder prepared with 7 milling cycles at room temperature to replace up to 5 wt.% polyol has the best macroscopic quality, with the resilience 50.08% and the compression set 7.2%, which is similar to the original F-PUF. Moreover, it is found that increasing milling cycles can reduce the particle size of the powder, and leads to a slight decrease in the density of F-PUF containing powder. Although the increased content of powder will lead to a decrease in resilience and an increase in compression of the F-PUF products, the F-PUF containing powder prepared with 7 milling cycles at room temperature to replace 15 wt.% polyol has a density similar to the original F-PUF, with resilience 49.08% and compression set 7.8%, which is still within the range of product requirements, indicating that the recycling method can be one of the most potentially environmental ways for high-value recycling of F-PUF scraps in industrial production. In the future, a quantitative analysis of the products generated due to chemical bonds broken and induced by mechanochemical effects will be studied.

## Figures and Tables

**Table 1:** Preparation formula of the F-PUF containing powder.

Component	Powder-Polyol Mixture	TDI	A33	D19	L618	A1	MC	Deionized Water
Mass Fraction (phr)	100	32.8	0.3	0.17	1.2	0.05	10	2.2

**Table 2:** VMD and particle temperature of the powder.

Experiment Number	Milling Cycles	Roller Temperature	VMD μm	Particle Temperature °C
1	3	Room temperature	165.58 ± 2.58	70.0 ± 0.4
2	5	Room temperature	103.15 ± 2.33	76.1 ± 0.2
3	7	Room temperature	97.73 ± 2.06	79.3 ± 0.3
4	9	Room temperature	76.82 ± 3.06	78.0 ± 0.3
5	7	40 °C	97.14 ± 2.13	80.5 ± 0.4
6	7	60 °C	94.87 ± 1.55	85.5 ± 0.2
7	7	80 °C	92.16 ± 1.03	93.2 ± 0.2

**Table 3:** Macro quality of the F-PUF containing powder and original F-PUF.

Sample	Powder Content in Powder-Polyol Mixture%	Density Kg/m^3^	Resilience %	Compression Set %
Powder prepared in Experiment 1	5	26.70 ± 0.21	48.46 ± 0.30	7.4 ± 0.3
	10	26.14 ± 0.17	48.25 ± 0.41	7.7 ± 0.2
	15	28.32 ± 0.24	47.43 ± 0.32	7.9 ± 0.4
	20	28.72 ± 0.22	42.74 ± 0.43	8.4 ± 0.3
Powder prepared in Experiment 3	5	26.48 ± 0.12	50.08 ± 0.24	7.2 ± 0.4
	10	25.12 ± 0.21	49.22 ± 0.22	7.5 ± 0.2
	15	25.26 ± 0.23	49.08 ± 0.27	7.8 ± 0.3
	20	25.47 ± 0.22	43.50 ± 0.33	8.2 ± 0.2
Powder prepared in Experiment 7	5	26.50 ± 0.18	50.43 ± 0.43	7.3 ± 0.3
	10	26.10 ± 0.13	49.17 ± 0.25	7.7 ± 0.5
	15	25.80 ± 0.10	48.56 ± 0.42	7.9 ± 0.2
	20	25.26 ± 0.13	43.53 ± 0.35	8.3 ± 0.5
original F-PUF	0	24.00 ± 0.20	50.00 ± 0.32	7.2 ± 0.2

## Data Availability

Not applicable.
